# Mental Health in Spanish Veterinarians: Emotional Exhaustion, Affective Symptomatology, and Suicidal Ideation

**DOI:** 10.3390/ejihpe16040049

**Published:** 2026-03-31

**Authors:** Sergio Guntín, Santiago López-Roel, Manuel Isorna, Francisca Fariña

**Affiliations:** 1 Forensic Psychology Unit, University of Santiago de Compostela, 15782 Santiago de Compostela, Spain; sergioguntin.mareque@usc.es; 2 Department of Psychology, University of A Coruña, 15008 A Coruña, Spain; s.lopez.roel@udc.es; 3 UNESCO Chair in Transformative Education, University of Vigo, 36310 Vigo, Spain; francisca@uvigo.es

**Keywords:** veterinarian, veterinary clinical assistant, occupational health, burnout, emotional exhaustion, stress, depression, anxiety, suicide, prevention

## Abstract

The mental health of veterinary professionals has become an increasing concern due to the high levels of psychological distress and suicide risk reported in this profession. The present study examined the association between emotional exhaustion and suicidal ideation, considering the mediating role of depressive, anxiety, and stress symptoms. A total of 216 Spanish veterinary professionals completed standardized questionnaires assessing emotional exhaustion, affective symptoms, and suicidal ideation. The results revealed a high prevalence of emotional exhaustion, with more than half of the participants reporting high or very high levels. In addition, a considerable proportion of the sample reported experiencing at least one indicator of suicidal ideation during the previous year. Emotional exhaustion was associated with higher levels of depression, anxiety, and stress. However, the mediation analysis indicated that only depressive symptoms explained the relationship between emotional exhaustion and suicidal ideation, whereas anxiety and stress did not show a significant mediating effect. Overall, the model accounted for a substantial proportion of the variance in suicidal ideation. These findings highlight the central role of depression in the link between emotional exhaustion and suicidal ideation, underscoring the need for early detection and prevention of depressive symptoms in veterinary professionals.

## 1. Introduction

In recent years, the mental health of veterinary professionals has become an area of growing interest and concern in the international scientific literature (Neubauer et al., 2024). Evidence indicates a high prevalence of psychological distress in this group (Cordova et al., 2025), significantly higher than that observed in the general population (Tomasi et al., 2019), and which may already emerge during university training (Schunter et al., 2025).

### 1.1. Mental Health Problems and Suicide Risk in Veterinary Professionals

Veterinary practice involves continuous exposure to specific psychosocial demands that increase professionals’ emotional vulnerability (Felipe, 2025). Several studies have documented that veterinarians face multiple stressors that negatively affect their mental well-being (Podpecan et al., 2025), within a particularly demanding occupational, socioeconomic, and cultural context (Jansen et al., 2024). These factors include work overload, constant ethical pressure, professional isolation, and limited social recognition, conditions that may foster frustration, depersonalization, and severe affective symptomatology, potentially leading to burnout processes, compassion fatigue, and secondary traumatic stress (Felipe, 2025; da Silva et al., 2023). Consistent with this, empirical evidence shows that veterinary professionals exhibit particularly high rates of anxiety, depression, emotional exhaustion, and suicidal ideation, frequently exceeding those reported in other healthcare professions (Bartram et al., 2009b; Best et al., 2020; Hatch et al., 2011; Hernández-Esteve et al., 2025; Máté et al., 2025). Mental health problems and suicide risk also affect veterinary students (Schunter et al., 2025).

In the United Kingdom, Bartram et al. (2009a) found that veterinarians scored significantly lower on general psychological well-being and reported higher levels of psychopathology compared with the general population. Similarly, international studies have shown that burnout levels in this group exceed those of the general population (Pohl et al., 2022). Research conducted in Austria (Neubauer et al., 2024), Australia (Hatch et al., 2011), and Canada (Best et al., 2020) has confirmed a high prevalence of burnout and depression among veterinarians, as well as overall mental health impairment, with particularly high rates among women. From this perspective, the literature suggests that gender may play a relevant role in the experience and expression of psychological distress within the veterinary profession; however, findings regarding gender differences in suicidal ideation are inconclusive and appear to depend on context and the specific indicators assessed (Neubauer et al., 2024; Perret et al., 2020; Schwerdtfeger et al., 2024). In a European study examining veterinarians’ mental health status, Jansen et al. (2024) reported substantial between-country differences; however, overall stress levels and the need for sick leave due to diminished mental well-being were high, while overall well-being was low. They also found that early-career veterinarians and female professionals were at greater risk of mental health deterioration. Age and years of professional experience have likewise been identified as relevant variables, with some studies suggesting greater vulnerability to psychological distress during early career stages, whereas among more experienced professionals, cumulative strain or healthy worker selection effects may operate—individuals with poorer mental health may leave the profession earlier, while those who remain tend to be more resilient—indicating potentially non-linear associations between professional experience and mental well-being (Jansen et al., 2024; Neubauer et al., 2024; Schunter et al., 2025). Overall, mental health problems and suicide risk among veterinary professionals are well documented (da Silva et al., 2023; Peixoto, 2025; Schunter et al., 2025).

Suicide within the veterinary profession represents one of the most alarming indicators of mental health deterioration in this group. Scientific evidence indicates that veterinarians are at greater risk of suicide than the general population (Erbe, 2024), with rates reported to be up to four times higher in countries such as Australia (Smith & Hawgood, 2025). In North America, Tomasi et al. (2019) retrospectively analyzed the deaths of 11,620 veterinarians between 1979 and 2015, finding that 3.42% were due to suicide, with a significant overrepresentation of women, who were between 3.4 and 5 times more likely to die by suicide compared with women in the general population. Consistently, Nett et al. (2015) reported that 17% of U.S. veterinarians had experienced suicidal ideation during their professional careers and that 1% had made at least one suicide attempt. Similarly, Peixoto (2025) found that 3.5% of Portuguese veterinarians had attempted suicide at least once in their lifetime.

### 1.2. Explanatory Framework: Emotional Exhaustion and Psychological Distress

From an explanatory perspective, psychological distress in veterinarians can be understood as the result of interactions among individual, organizational, and contextual factors. At the individual level, clinical perfectionism has been identified as a relevant risk factor, as it intensifies moral distress and reduces coping capacity in the face of occupational and ethical conflicts (Lewis & Cardwell, 2020; Crane et al., 2015). At the organizational level, the literature highlights structural factors such as lack of institutional support, imbalance between job demands and resources, professional isolation, economic pressures, and work–life imbalance (Peterson et al., 2018; Bartram et al., 2009b; Dalum et al., 2022). Repeated exposure to ethical dilemmas, such as performing euthanasia in unwanted contexts or facing treatment limitations due to financial constraints, significantly increases emotional exhaustion (Moses et al., 2018). In addition, easy access to lethal means, such as anesthetics, opioids, and barbiturates, has been identified as a critical factor in veterinary suicide (Witte et al., 2019), reinforcing the relevance of means-restriction-based preventive strategies (Hawton, 2007; Yip et al., 2012). Finally, emotional overinvolvement with patients and clients may foster professional self-sacrificial dynamics that, despite increasing client satisfaction, may occur at the cost of greater psychological deterioration for the professional (Perret et al., 2020).

Emotional exhaustion, commonly defined as a state of feeling emotionally overextended and depleted of emotional resources as a result of prolonged work-related stress (Maslach et al., 2001), constitutes the core dimension of burnout. From the theoretical perspective of psychosocial work stressors, it can be conceptualized as a relatively distal occupational risk factor that emerges as a sustained response to adverse working conditions (e.g., high demands, low control, effort–reward imbalance, job insecurity, and low social support), which are recognized psychosocial determinants of health and potentially modifiable (Karasek, 1979; Siegrist, 2002; De Witte, 2005; Milner et al., 2018).

Within this framework, depressive, anxiety-based, and perceived stress symptoms may be understood as more proximal vulnerability factors that increase the likelihood of suicidal ideation. Thus, the association between emotional exhaustion and suicidal ideation is theoretically more plausible when exhaustion is accompanied by deteriorating mental health and heightened psychological distress, given the well-established links between occupational stressors and common mental disorders such as depression (Bonde, 2008; Netterstrøm et al., 2008).

In line with this formulation, evidence suggests that psychosocial job stressors are associated with increased risk of suicidality (Milner et al., 2018), while a large body of research supports the role of mental health problems as key contributors to suicidal ideation and behavior (Arsenault-Lapierre et al., 2004). However, the specific mechanisms linking occupational stressors and suicidality remain complex and are likely to involve multiple intermediate processes, including psychological distress and mental health impairment.

At the same time, and consistent with methodological concerns regarding reverse causality in observational research, a plausible bidirectional relationship can be considered: (a) adverse working conditions may contribute to emotional exhaustion and increased depressive, anxiety, and perceived stress symptoms, thereby elevating suicidal ideation risk; and (b) affective symptomatology may bias the perception and reporting of occupational stressors or influence selection into jobs with lower psychosocial quality, intensifying exhaustion.

Finally, this stress–exhaustion–psychopathology–suicidal ideation framework is also compatible with biological mechanisms (e.g., allostatic load and alterations in neural circuits related to memory, fear/anxiety, and mood regulation), which may amplify the transition from chronic occupational stress to affective symptoms and suicidal cognitions (McEwen & Tucker, 2011).

Overall, the evidence suggests that emotional exhaustion is consistently associated with mental health deterioration among veterinary professionals and with increased risk of suicidal ideation. However, the psychological mechanisms underlying this association remain insufficiently clarified. In particular, depressive symptoms, anxiety, and perceived stress have been identified as potential intermediate processes that may help explain the relationship between emotional exhaustion and suicidal ideation.

### 1.3. Objectives

The present study aims to examine the association between emotional exhaustion and suicidal ideation, considering the mediating role of depression, anxiety, and perceived stress. More specifically, the study pursues the following objectives:

- To examine the association between emotional exhaustion and levels of depression, anxiety, and perceived stress.
- To analyze whether depression, anxiety, and perceived stress act as mediating variables in the relationship between emotional exhaustion and suicidal ideation.
- To identify which of these emotional variables most significantly explains this mediation.
- To explore the extent to which affective variables account for the relationship between emotional exhaustion and suicidal ideation, distinguishing between total and partial mediation.

## 2. Materials and Methods

### 2.1. Sample

The sample was recruited from August to September 2025 in collaboration with the Galician Veterinary Business Association (EVGal) and the collaborative alliance EmpreVet, which includes the Málaga Veterinary Business Association (AEMAVE), the Veterinary Business Association of Aragón (AEVAR), the Association of Clinical Veterinary Businesses of Sevilla (CEVE Sevilla), and EVGal. Recruitment also involved disseminating the questionnaire within an online community restricted to licensed veterinary professionals. An online survey was administered via Microsoft Forms. Participation was voluntary, and no compensation was provided.

The final sample consisted of 216 participants, 168 women and 48 men, aged between 22 and 65 years (*M* = 43.19, *SD* = 9.945). Of these, 189 were clinical veterinarians and 27 were veterinary clinical assistants, representing 14 autonomous communities in Spain. Regarding employment status, 106 held managerial positions in a clinic, while 110 were employed as salaried staff members.

In terms of sex distribution, representativeness could only be evaluated for the subgroup of clinical veterinarians, as official statistics in Spain report sex distribution exclusively for this professional category. Within this subgroup (*n* = 189), the sample did not fully reflect the national distribution. According to data from the National Statistics Institute (Instituto Nacional de Estadística, n.d.), women represent 54.4% of clinical veterinarians in Spain, whereas in the present study they accounted for 75.7%. Conversely, men were underrepresented (24.3% in the sample vs. 45.6% in the population). These figures are based on official statistics derived from registered (i.e., licensed) veterinarians, which constitute the closest available proxy for the active professional population.

Descriptive analyses indicated that women in the sample were younger on average (*M* = 41.40, *SD* = 9.56) than men (*M* = 49.46, *SD* = 8.75). Most participants in both groups were clinical veterinarians (143 women and 46 men), with a smaller proportion of veterinary clinical assistants. In terms of employment position, both managerial and employed roles were represented in each group, with women more frequently represented in absolute numbers due to their larger sample size (Table 1).

### 2.2. Measures

The following psychometric instruments were used to assess the study variables:

- Maslach Burnout Inventory—General Survey (MBI-GS; Maslach et al., 1996; Schaufeli et al., 1996; Salanova et al., 2000). This instrument assesses burnout across different occupations as a multidimensional construct including emotional exhaustion, cynicism, and professional efficacy. In the present study, the focus was placed on the emotional exhaustion dimension, as it is considered the core component of burnout (Maslach & Leiter, 2016; Seidler et al., 2014) and is closely associated with psychological distress (Koutsimani et al., 2019). In addition, previous research has shown that even brief measures of emotional exhaustion, including single-item approaches, yield associations with key outcomes similar to those obtained using the full MBI (West et al., 2012; Dolan et al., 2015). The emotional exhaustion subscale consists of 5 items (e.g., “I feel emotionally drained from my work”), rated on a 7-point Likert scale ranging from 0 (“Never”) to 6 (“Always”). Scores were computed as the mean of the items. The Spanish version has shown adequate reliability and validity in working populations. In a sample of Spanish workers, Cronbach’s alpha coefficients were 0.85 for emotional exhaustion, 0.78 for cynicism, and 0.73 for professional efficacy (Salanova et al., 2000). Similar or higher reliability coefficients have been reported in larger heterogeneous samples of Spanish workers, with alpha values of 0.87, 0.85, and 0.78 for the three dimensions, respectively (Bresó et al., 2006).
- Depression Anxiety Stress Scales (DASS-21; Lovibond & Lovibond, 1995; Daza et al., 2002). This instrument measures symptoms of depression, anxiety, and stress experienced during the past week and consists of 21 items distributed across three subscales. Example items include “I couldn’t seem to experience any positive feeling at all”, “I felt scared without any good reason”, and “I found it difficult to relax”. Each item is rated on a 4-point Likert scale ranging from 0 (“Did not apply to me at all”) to 3 (“Applied to me very much or most of the time”), yielding a theoretical subscale score range of 0–42 after multiplying summed scores by two to ensure comparability with the original DASS-42. Higher scores indicate greater symptom severity in each dimension. The Hispanic version has shown a clear three-factor structure and excellent internal consistency, with Cronbach’s alpha coefficients of 0.93 for depression, 0.86 for anxiety, and 0.91 for stress (Daza et al., 2002). Similar psychometric properties have been reported in Spanish samples (Bados et al., 2005; Ruiz et al., 2017).
- Paykel Suicide Scale (PSS; Paykel et al., 1974). This scale assesses the presence and intensity of suicidal ideation during the past year through a brief set of questions addressing thoughts of self-harm and suicide planning. The first part evaluates the presence of suicidal ideation using a dichotomous response format (“Yes”/“No”), yielding a total score ranging from 0 to 5, with higher scores indicating greater presence of suicidal ideation or behavior. An example item is: “Have you ever wished you were dead (for example, going to sleep and wishing you would not wake up)?” If affirmative responses are endorsed, a second section assesses the intensity of suicidal ideation using a 7-point Likert-type scale ranging from 1 (“Very rarely”) to 7 (“Very frequently”). The PSS has shown adequate psychometric properties in Spanish samples, including a unidimensional structure and good internal consistency across different populations, with reliability coefficients ranging approximately from 0.76 to 0.92 (Fonseca-Pedrero & Pérez de Albéniz, 2020; Martínez-Galiano et al., 2024; Pinazo-Clapés et al., 2025).

All variables were operationalized as dimensional self-reported scores derived from validated instruments and do not constitute clinical diagnoses. For statistical analyses, raw summed scores were used; any score transformations described above were applied solely for normative or interpretative purposes.

### 2.3. Data Analysis

After cleaning the dataset, descriptive analyses were conducted for all variables included in the study (frequencies, means, standard deviations, among others). Pearson correlation analyses (*r*) and mean comparisons (Student’s *t* tests and ANOVA with Tukey post hoc tests) were then performed to examine potential differences across key socio-demographic and reference variables, including sex, age, profession, and professional role. Effect sizes were reported for all analyses. Pearson correlations (*r*) were interpreted according to Cohen’s (1988) criteria (small: 0.10, medium: 0.30, large: 0.50). For mean comparisons, Hedges’ g was used and interpreted following Cohen’s (1988) guidelines (small: 0.20, medium: 0.50, large: 0.80). For ANOVA analyses, omega squared (ω^2^) was reported and interpreted using conventional benchmarks analogous to those proposed by Cohen (1988) for effect size interpretation (small: 0.01, medium: 0.06, large: 0.14).

Finally, a multiple mediation model with three parallel mediators was estimated using the PROCESS macro (version 4.2; Hayes, 2022) in IBM SPSS Statistics, version 29.0.1.0 (171). PROCESS Model 4 was applied with 10,000 bootstrap samples, and the HC3 correction was used for the estimation of standard errors. Emotional exhaustion was specified as the independent (predictor) variable, suicidal ideation as the dependent variable, and depression, anxiety, and stress as potential mediators. For this analysis, each scale score was computed by summing the scores of all items comprising the respective measure. Standardized regression coefficients (*β*) were reported as indicators of effect size and interpreted according to conventional guidelines (Cohen, 1988), with values of approximately 0.10, 0.30, and 0.50 considered small, medium, and large effects, respectively. Prior to estimating the mediation model, collinearity diagnostics were performed to assess potential multicollinearity among predictors.

## 3. Results

### 3.1. Exploratory Analyses

In addition to the primary objective of examining the mediating role of affective symptoms, a set of exploratory analyses was conducted to describe the study variables, assess their preliminary associations, and determine the statistical suitability of the mediation model. Table 2 presents the descriptive statistics for the scales used. As shown, skewness and kurtosis values fell within acceptable ranges (±1). In terms of internal consistency, all scales demonstrated adequate reliability according to Nunnally’s (1978) criteria, with Cronbach’s α coefficients ranging from 0.80 to 0.94 and McDonald’s ω coefficients ranging from 0.85 to 0.94.

The distribution of scores for Emotional Exhaustion shows a clear shift toward the upper ranges of the MBI-GS (Table 3). More than half of the participants fall within the high or very high levels (61.1%, *n* = 132), indicating a notable presence of symptoms consistent with significant emotional exhaustion. In contrast, only one quarter of the sample (25.4%, *n* = 55) scored within the very low, low, or medium–low ranges, which are typically associated with relatively unaffected functioning.

Severity levels on the Depression, Anxiety, and Stress subscales of the DASS-21 (Table 4) indicate that 36.1% of participants (*n* = 78) fell within the normal range for both depression and anxiety, while 43.5% (*n* = 94) did so for stress. Moderate levels were more frequent for depression (28.2%, *n* = 61) and anxiety (28.7%, *n* = 62); however, for stress, a higher proportion of cases were classified as mild rather than moderate (21.8%, *n* = 47). Severe and extremely severe levels were less prevalent overall, with anxiety showing the highest proportion of extremely severe cases (13.4%, *n* = 29), followed by depression (9.3%, *n* = 20) and stress (4.2%, *n* = 9).

Finally, the mean score observed for the PSS indicates that a considerable proportion of the sample reported more than one type of suicidal thought or behavior (*M* = 1.28, *SD* = 1.55). Specifically, 40.5% of participants (*n* = 107) endorsed at least one of the five PSS items, with 27.8% (*n* = 60) scoring on three or more of the five indicators. At the item level, 41.7% (*n* = 90) reported having felt during the past year that “life is not worth living.” Additionally, 32.9% (*n* = 71) reported having wished to be dead, and 35.2% (*n* = 76) reported having thought about taking their own life during the same period. A total of 15.7% (*n* = 34) indicated that they had seriously considered and planned this possibility, while 2.8% (*n* = 6) reported having made at least one suicide attempt.

Regarding the correlation analyses (Table 5), all study variables were positively and significantly correlated with one another (*p* < 0.001). Emotional exhaustion (EMO) showed moderate associations with depression (*r* = 0.60), anxiety (*r* = 0.60), and stress (*r* = 0.66), suggesting a link between occupational strain and psychological distress. Suicidal ideation (SUI) was also significantly associated with all variables, with the strongest correlations observed for depression (*r* = 0.64) and stress (*r* = 0.52).

Regarding group comparisons by sex, statistically significant differences were identified only for anxiety (*t*[214] = 2.95, *p* < 0.05, *g* = 0.48, 95% CI [0.16, 0.80]), with women reporting higher mean scores than men. The effect size for this difference was moderate. No statistically significant differences were found for emotional exhaustion (*t*[214] = 1.80, *p* = 0.07, *g* = 0.29, 95% CI [−0.03, 0.61]), stress (*t*[214] = 1.87, *p* = 0.06, *g* = 0.31, 95% CI [−0.02, 0.63]), depression (*t*[214] = 1.10, *p* = 0.27, *g* = 0.18, 95% CI [−0.14, 0.50]), or suicidal ideation (*t*[214] = 0.06, *p* = 0.95, *g* = 0.01, 95% CI [−0.31, 0.33]). Effect sizes for these non-significant comparisons ranged from negligible to small, and confidence intervals included the value zero, which reflects uncertainty regarding their magnitude and direction.

Analyses also revealed differences by profession. Veterinary clinical assistants reported significantly lower levels of emotional distress than clinical veterinarians in emotional exhaustion (*t*[214] = 2.60, *p* < 0.05, *g* = 0.53, 95% CI [0.13, 0.94]), anxiety (*t*[214] = 2.52, *p* < 0.05, *g* = 0.52, 95% CI [0.11, 0.92]), and stress (*t*[214] = 2.09, *p* < 0.05, *g* = 0.43, 95% CI [0.02, 0.83]). These differences were of moderate magnitude. No statistically significant differences were found for depression (*t*[214] = 1.96, *p* = 0.05, *g* = 0.40, 95% CI [−0.01, 0.81]) or suicidal ideation (*t*[214] = −0.45, *p* = 0.65, *g* = −0.09, 95% CI [−0.49, 0.31]). For these non-significant outcomes, effect sizes ranged from negligible to small to moderate, and the inclusion of the value zero in the confidence intervals indicates uncertainty about the existence and direction of the effects. Additionally, professional role (owner/manager vs. employee) was not significantly associated with any of the variables analyzed (*p* > 0.10 in all cases).

**Figure 1:**
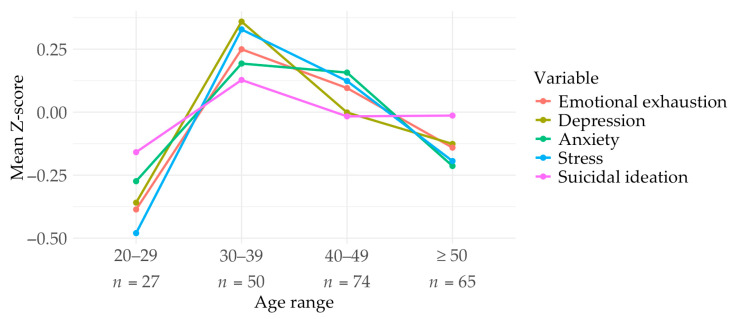
Comparison of all variables across age groups. Note. N = 216. n = sample size of each group. Scores for each variable have been standardized to aid interpretation.

Age-group comparisons showed differences across age groups (Figure 1), with participants aged 30–39 years consistently reporting higher scores across all variables. However, these differences were generally small in magnitude and not uniformly supported by statistically significant pairwise comparisons. Significant group differences were found for emotional exhaustion (*F*[3, 212] = 3.13, *p* < 0.05, ω^2^ = 0.01 [0, 0.03]), with the 30–39 age group scoring higher than the 20–29 group (*M*_diff_ = 4.69, *p* < 0.05). Significant age differences also emerged for depression (*F*[3, 212] = 3.80, *p* < 0.05, ω^2^ = 0.01 [0, 0.03]) and stress (*F*[3, 212] = 5.37, *p* = 0.001, ω^2^ = 0.02 [0, 0.04]). For depression, the 30–39 group scored significantly higher than both the 20–29 (*M*_diff_ = 3.30, *p* < 0.05) and 50–65 groups (*M*_diff_ = 2.23, *p* < 0.05). For stress, the 30–39 group also scored significantly higher than the 20–29 (*M*_diff_ = 3.08, *p* < 0.05) and 50–65 groups (*M*_diff_ = 2.00, *p* < 0.05). Additionally, significant differences in stress were found between the 40–49 and 20–29 groups (*M*_diff_ = 2.30, *p* < 0.05), with higher scores in the former. Effect sizes were small across significant comparisons, suggesting that age accounted for a limited proportion of variance in these outcomes. Age differences were also observed for anxiety (*F*[3, 212] = 2.97, *p* < 0.05, ω^2^ = 0.01 [0, 0.03]); however, adjusted post hoc comparisons did not identify significant pairwise differences between groups. In contrast, no significant age differences were found for suicidal ideation (PSS; *F*[3, 212] = 0.51, *p* > 0.05).

Overall, the results reveal a notable presence of suicidal ideation at varying levels and a consistent pattern of associations between suicidal ideation and multiple indicators of psychological distress. Higher levels of emotional distress were observed among clinical veterinarians compared with veterinary clinical assistants, as well as among professionals aged 30 to 39 years compared with other age groups.

With respect to sex, statistically significant differences were found only for anxiety, with women reporting higher scores than men. Finally, no differences were identified in any of the study variables according to professional role (owner/manager vs. employee).

The high proportion of participants scoring outside the normal range suggests a pattern warranting particular attention, as individuals reporting elevated levels of symptomatology outnumber those reporting no psychological distress.

### 3.2. Multiple Mediation Analysis

**Figure 2:**
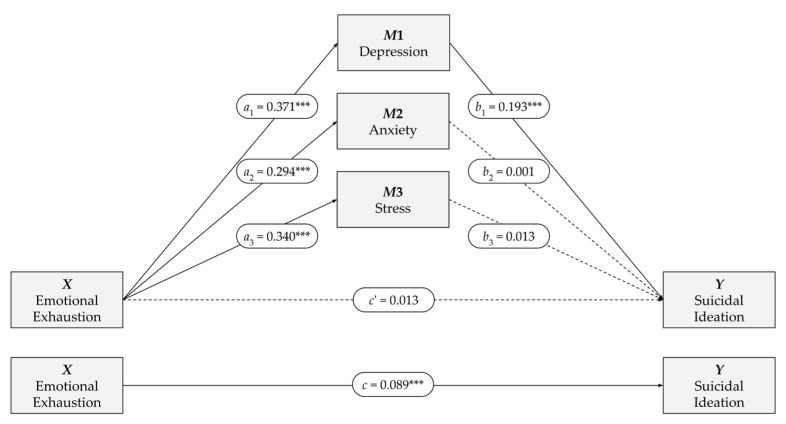
Parallel multiple mediation model between emotional exhaustion and suicidal ideation. Note. Parallel multiple mediation model (PROCESS, Model 4). The *a* paths represent the effects of the *X* variable on each mediator; the *b* paths represent the effects of each mediator on *Y*; *c* is the total effect of *X* on *Y*, and *c’* is the direct effect. Unstandardized coefficients are reported; for standardized coefficients, see Table 6 and Table 7. Solid lines indicate significant effects; dashed lines indicate non-significant effects. *** *p* < 0.001.

As shown in Figure 2, the independent variable *X* (emotional exhaustion) was a significant predictor of each of the three mediating variables: depression (*B* = 0.37, *p* < 0.001), anxiety (*B* = 0.29, *p* < 0.001), and stress (*B* = 0.34, *p* < 0.001). However, among these three variables, only depression (*M*1) was significantly associated with suicidal ideation (*B* = 0.19, *p* < 0.001), whereas anxiety (*M*2), stress (*M*3), and emotional exhaustion (*X*) were not significant predictors when included simultaneously in the model (Table 6). Collinearity diagnostics indicated acceptable levels of multicollinearity among predictors (VIFs ranging from 1.86 to 3.69), supporting the stability of the regression estimates.

In this sense, emotional exhaustion was associated with a 0.37-point increase in depression for each one-unit increase in exhaustion, and depression, in turn, was associated with a 0.19-point increase in suicidal ideation for each one-unit increase in depression. In standardized terms, both effects were substantial (*β* = 0.60 and *β* = 0.57). This model accounted for 40.9% of the total variance in the dependent variable (suicidal ideation).

The direct effect was not statistically significant (Table 7; *B* = 0.01, *p* = 0.45), indicating that emotional exhaustion (*X*) does not predict suicidal ideation (*Y*) when the three mediating variables (*M*1, *M*2, and *M*3) are included in the model. However, emotional exhaustion significantly predicted suicidal ideation in the total effect model (Table 7; *B* = 0.10, *p* < 0.001), that is, when the mediating variables were not included. The fact that the association between emotional exhaustion and suicidal ideation becomes non-significant when the mediators are introduced is consistent with statistical full mediation within the tested model, with depression (M1) accounting for the indirect effect.

Regarding the indirect effects (Table 7), a significant overall indirect effect was observed (*B* = 0.08, 95% CI [0.05, 0.10]). However, depression (*M*1) was the only relevant mediator of the relationship between emotional exhaustion (*X*) and suicidal ideation (*Y*), accounting for virtually the entire indirect effect (*B* = 0.07, 95% CI [0.05, 0.09]). In contrast, anxiety and stress (*M*2 and *M*3) did not play a mediating role.

In summary, a positive and significant association was observed between emotional exhaustion and depression, anxiety, and stress; however, only depression was directly and significantly associated with suicidal ideation. The total effect of emotional exhaustion on suicidal ideation was positive and significant, but it became non-significant when the mediators were included in the model. The bootstrap analysis confirmed that depression was the only significant mediator. Neither anxiety nor stress exerted a mediating effect when included simultaneously with depression.

## 4. Discussion

Overall, the results of this study, in line with previous research (Cordova et al., 2025; Máté et al., 2025; Perret et al., 2020; Podpecan et al., 2025; da Silva et al., 2023; Schunter et al., 2025; Tomasi et al., 2019), indicate that emotional exhaustion, psychological distress, and suicidal ideation among veterinary professionals constitute a relevant problem that should be addressed through comprehensive approaches.

Mental health problems and burnout among veterinary professionals have consistently been reported as more prevalent among women (Neubauer et al., 2024; Osca et al., 2024; Peixoto, 2025; Perret et al., 2020; San Martín et al., 2023). In line with this evidence, the present findings show sex differences in anxiety levels. However, contrary to the results reported by Schwerdtfeger et al. (2024), no significant sex differences were found in suicidal ideation, which is consistent with findings reported by Perret et al. (2020) in veterinary students. This may be partly explained by evidence suggesting that female veterinarians are more likely than their male counterparts to seek professional support when needed (Máté et al., 2025).

The findings suggest that psychological distress may vary across the lifespan among veterinary professionals, although these age-related differences were small in magnitude. Higher scores were generally observed among individuals aged 30 to 39 years; however, this pattern accounted for only a limited proportion of variance. This period may represent a stage of increased emotional vulnerability, characterized by growing professional and personal responsibilities, reflected in higher levels of emotional exhaustion, depression, and stress (Fritschi et al., 2009). In contrast, no meaningful age-related differences were found in suicidal ideation. It should nevertheless be considered that Máté et al. (2025) reported that older veterinarians tend to manage their mental health more effectively and are more likely to seek help when needed.

Clinical veterinarians reported higher levels of emotional exhaustion, anxiety, and stress than veterinary clinical assistants, which may reflect the greater emotional demands typically associated with clinical practice, including decision-making responsibilities, patient care, and interactions with owners. In contrast, no differences were found in depression or suicidal ideation, which contrasts with the findings of Milner et al. (2018), who reported lower suicide risk among veterinary nurses compared with clinical veterinarians.

Professional role (owner vs. employee) did not show a significant impact on psychological distress. This finding may suggest that the emotional demands associated with veterinary clinical work are present across hierarchical levels. Nevertheless, it cannot be ruled out that practice owners may be more likely to manage their mental health effectively and seek support when necessary (Máté et al., 2025).

Taken together, these findings highlight the need to implement preventive mental health strategies tailored to different career stages and professional roles within the veterinary field.

Regarding the association between emotional exhaustion and suicidal ideation, as well as the mediating role of depression, anxiety, and perceived stress, the present findings provide evidence of potential explanatory processes associated with suicidality among veterinary professionals. Consistent with previous research in healthcare professionals showing that the relationship between burnout and suicidal ideation weakens or becomes non-significant when depressive symptoms are controlled (Menon et al., 2020), the results suggest that emotional exhaustion is robustly associated with general affective distress, but its link with suicidal ideation is primarily accounted for by depressive symptoms. From a vulnerability–stress perspective, these findings are consistent with the interpretation that emotional exhaustion may constitute a distal occupational stressor associated with greater depressive vulnerability, the latter representing the more proximal correlate of suicidal ideation within this model.

In addition, emotional exhaustion was positively and significantly associated with higher levels of depression, anxiety, and stress, consistent with previous studies identifying burnout as a chronic stressor capable of eroding psychological well-being among veterinary professionals (Bartram et al., 2009b; Hatch et al., 2011; Pohl et al., 2022). These findings further support the conceptualization of emotional exhaustion as a cross-cutting vulnerability factor associated with affective symptomatology, particularly in work contexts characterized by high emotional demands, persistent ethical pressures (e.g., euthanasia decision-making, conflicts with pet owners regarding treatment limitations due to financial constraints), and limited resources (Felipe, 2025; Moses et al., 2018; da Silva et al., 2023).

Importantly, emotional exhaustion did not directly predict suicidal ideation when affective symptoms were simultaneously considered. Although the total effect of emotional exhaustion on suicidal ideation was significant, this effect became non-significant once the mediators were introduced, indicating that the association is not direct but is statistically accounted for by intermediate psychological variables. These findings indicate that emotional exhaustion, in itself, is not independently linked to suicidal ideation, but rather appears to be associated with it through co-occurring depressive symptomatology.

The multiple mediation analysis further indicated that depression was the only significant mediator in the relationship between emotional exhaustion and suicidal ideation, accounting for virtually the entire indirect effect. This finding is consistent with previous research identifying depression as one of the most robust predictors of suicidal ideation among veterinary professionals (Nett et al., 2015; Tomasi et al., 2019; Dalum et al., 2022) and as a primary risk factor for suicidal ideation and suicide attempts (da Silva et al., 2023). In contrast, neither anxiety nor perceived stress showed a significant mediating effect when included simultaneously with depression. Although both variables were positively associated with emotional exhaustion and suicidal ideation at the correlational level, their explanatory contribution became non-significant after controlling for depressive symptoms. These results suggest that, while anxiety and stress are components of general psychological distress, they do not appear to play a central role in the association between emotional exhaustion and suicidal ideation. This may be partly attributable to the conceptual and empirical overlap among the DASS-21 subscales, despite acceptable levels of multicollinearity, which could have led depression to account for most of the mediating effect when all three dimensions were entered simultaneously in the model. Such a configuration may reflect the presence of a general factor of psychological distress underlying the dimensions of the DASS-21, with depressive symptomatology accounting for the largest proportion of variance specifically associated with suicidal ideation. Similar patterns have been reported in occupational research contexts, where depression emerges as the affective component most directly linked to hopelessness, loss of meaning, and self-harm ideation (Milner et al., 2018; da Silva et al., 2023).

### 4.1. Implications

The results of this study have several implications, both theoretical and practical, particularly in relation to the design of intervention programs in the veterinary context.

First, at a theoretical level, this study contributes to understanding the interaction between emotional exhaustion and suicidal ideation, highlighting the role of depressive symptomatology in comparison to other forms of psycho-emotional distress, all integrated into a single theoretical model.

At a more applied level, and considering the findings obtained, depressive symptomatology emerges as a particularly relevant indicator associated with emotional exhaustion in the workplace. Thus, conducting a systematic assessment of depressive symptoms could help identify more vulnerable profiles, enabling the implementation of interventions specifically tailored to this group and improving the allocation of support resources.

On the other hand, the fact that anxiety and stress did not play a mediating role in the model suggests that measures focused exclusively on these types of manifestations may be insufficient for preventing suicidal ideation. Therefore, mental health promotion programs could benefit from including specific components aimed at the detection and treatment of depressive symptomatology.

Furthermore, high levels of emotional exhaustion and affective distress may require the adoption of sustained preventive measures, beyond isolated or reactive interventions. Although this study did not directly assess organizational variables, the relationship between profession-related emotional exhaustion and suicidal ideation highlights workplace characteristics as a potential factor involved in the onset and maintenance of psychological distress, as supported by the literature.

Finally, it should be noted that these implications must be considered within the limits of a correlational design and the study’s own limitations, despite providing a solid foundation for guiding future preventive actions and new lines of research.

### 4.2. Limitations

Regarding the limitations of the present study, it is important to note, first, that the results described above reflect associations between variables and, due to the cross-sectional correlational design, do not allow for causal inferences. Furthermore, the absence of temporal measurements prevents verification of temporal precedence among emotional exhaustion, affective symptoms, and suicidal ideation.

Second, the representativeness of the sample with respect to the target population cannot be assumed. Although participants were drawn from different autonomous communities and presented diverse sociodemographic profiles, the generalizability of the findings remains limited. A related limitation concerns the potential response bias associated with self-selection. Thus, it is possible that individuals experiencing higher levels of emotional distress were more motivated to participate in the study and even at the time of completing the questionnaire.

Lastly, the inherent limitations of the psychometric instruments used should be considered. Their design, underlying assumptions, and measurement constraints may influence how results are obtained and interpreted. It is also essential to emphasize that the scores derived from these instruments do not, in themselves, constitute a clinical diagnosis.

### 4.3. Future Research

Building on the present findings, future research should further examine the mechanisms underlying the relationship between emotional exhaustion, depressive symptoms, and suicidal ideation, clarifying the temporal and potentially causal relationships among these variables through longitudinal studies.

In addition, it would be advisable to analyze which combination of individual and contextual variables increases vulnerability to the deterioration of psycho-emotional well-being among veterinary professionals, examining the main static risk factors for predicting suicidal behavior, as well as dynamic factors focused on prevention and intervention.

It would also be important to investigate the extent to which training in coping skills and mental health promotion—both during veterinary education and throughout professional practice—could help reduce risk in this population. Exploring how these competencies may modulate the relationship between emotional exhaustion, depression, and suicide risk could represent an initial step toward addressing this issue.

## 5. Conclusions

The present study provides empirical evidence on the mental health of veterinary professionals in Spain, highlighting a high prevalence of emotional exhaustion, affective symptomatology, and suicidal ideation within this group. Emotional exhaustion was consistently associated with higher levels of depression, anxiety, and stress; however, only depressive symptoms emerged as a significant mediator in the relationship between emotional exhaustion and suicidal ideation. These findings support the conceptualization of emotional exhaustion as an occupational strain indicator associated with general affective distress, while depressive symptomatology appears to represent the more proximal correlate of suicidal ideation within the tested model.

From an applied perspective, the findings underscore the need for suicide prevention strategies in the veterinary profession to prioritize early detection and targeted intervention for depressive symptomatology, particularly among professionals experiencing high levels of emotional exhaustion. Interventions focused exclusively on stress management or burnout reduction may be insufficient if they are not integrated with systematic programs for psychological assessment, prevention, and treatment specifically addressing depression. The results also highlight the importance of incorporating training in psychological well-being promotion and effective management of emotional distress into veterinary education.

Overall, this study contributes to clarifying the pattern of associations linking occupational strain to suicidal ideation among veterinary professionals and emphasizes the importance of adopting comprehensive mental health approaches that address both organizational factors and individual emotional processes. Future longitudinal studies with larger samples will be necessary to further examine the directionality of these relationships and to evaluate the effectiveness of preventive interventions specifically designed to reduce the impact of emotional exhaustion and depression in this population.

## Figures and Tables

**Table 1:** Demographic and professional characteristics of the sample by sex.

Sex	*n*	Age			Professional Role		Employment Position	
		Mean	*SD*	Range	CV	VCA	Manager	Employed
Women	168	41.40	9.56	22–60	143	25	72	96
Men	48	49.46	8.75	25–65	46	2	34	14
Total	216	43.19	9.95	22–65	189	27	106	110

*N* = 216. *n* = Sample size of each group; *SD* = Standard deviation; CV = Clinical veterinarian; VCA = Veterinary clinical assistant.

**Table 2:** Descriptive statistics and reliability of the scales and subscales used in the study.

Variable	Mean	*SD*	Range	Skewness	Kurtosis	Cronbach’s α	McDonald’s ω
MBI-GS (EMO)	16.70	7.38	0–30	−0.07	−0.80	0.94	0.94
DASS-21 (DEP)	6.87	4.59	0–20	0.85	0.21	0.91	0.92
DASS-21 (ANX)	5.25	3.60	0–18	0.77	0.60	0.84	0.85
DASS-21 (STR)	8.57	3.81	0–19	0.46	0.04	0.87	0.87
PSS (SUI)	1.28	1.55	0–5	0.80	−0.75	0.81	0.86

*N* = 216. EMO = Emotional exhaustion; DEP = Depression; ANX = Anxiety; STR = Stress; SUI = Suicidal ideation; *SD* = Standard deviation; Range = Observed score range in the sample. All variables are reported as summed item scores, corresponding to the scores entered in the mediation model.

**Table 3:** Distribution of severity levels on the Emotional Exhaustion subscale of the MBI-GS.

MBI-GS	Normative Ranges	*n*	%
Very Low	<0.4	2	0.9
Low	0.5–1.2	21	9.7
Medium (Low)	1.3–2	32	14.8
Medium (High)	2.1–2.8	29	13.4
High	2.9–4.5	75	34.7
Very High	>4.5	57	26.4

*N* = 216. Scores for the Emotional Exhaustion scale were calculated by summing the responses to the five items corresponding to this dimension and dividing the total by 5, which is the number of items in the subscale. The resulting mean score was then classified according to the normative ranges shown in the table (Bresó et al., 2006).

**Table 4:** Severity distribution in Depression, Anxiety, and Stress (DASS-21).

DASS-21	Depression			Anxiety			Stress		
	Range	*n*	%	Range	*n*	%	Range	*n*	%
Normal	0–9	78	36.1	0–7	78	36.1	0–14	94	43.5
Mild	10–13	35	16.2	8–9	23	10.6	15–18	47	21.8
Moderate	14–20	61	28.2	10–14	62	28.7	19–25	41	19
Severe	21–27	22	10.2	15–19	24	11.1	26–33	25	11.6
Extremely Severe	>27	20	9.3	>19	29	13.4	>33	9	4.2

*N* = 216. Subscale scores were calculated by summing items within each subscale and multiplying by two to maintain comparability with the original 42-item version. The cut-off points applied were those recommended by the authors (Lovibond & Lovibond, 1995).

**Table 5:** Correlations coefficients (Pearson’s r) between the scales and subscales used in the study.

Variable	MBI-GS (EMO)	DASS-21 (DEP)	DASS-21 (ANX)	DASS-21 (STR)
DASS-21 (DEP)	0.60 ***			
DASS-21 (ANX)	0.60 ***	0.73 ***		
DASS-21 (STR)	0.66 ***	0.77 ***	0.79 ***	
PSS (SUI)	0.43 ***	0.64 ***	0.48 ***	0.52 ***

*N* = 216. EMO = Emotional exhaustion; DEP = Depression; ANX = Anxiety; STR = Stress; SUI = Suicidal ideation. *** *p* < 0.001.

**Table 6:** Regression models of mediation analysis.

Outcome	Predictor	*B*	*SE*	*t*	*p*	LLCI	ULCI	*β*	*R* ^2^
*M*1 (DEP)	*X* (EMO)	0.37	0.03	11.39	<0.001	0.31	0.44	0.60	0.36
*M*2 (ANX)	*X* (EMO)	0.29	0.03	10.58	<0.001	0.24	0.35	0.60	0.36
*M*3 (STR)	*X* (EMO)	0.34	0.03	12.35	<0.001	0.29	0.40	0.66	0.43
*Y* (SUI)	*X* (EMO)	0.01	0.02	0.76	0.45	−0.02	0.05	0.06	0.41
	*M*1 (DEP)	0.19	0.03	7.33	<0.001	0.14	0.25	0.57	
	*M*2 (ANX)	0.00	0.05	0.02	0.99	−0.09	0.09	0.00	
	*M*3 (STR)	0.01	0.04	0.30	0.76	−0.07	0.10	0.03	

EMO = Emotional exhaustion; DEP = Depression; ANX = Anxiety; STR = Stress; SUI = Suicidal ideation. *B* = Unstandardized coefficient; *SE* = heteroscedasticity-consistent standard error using the HC3 correction; *t* = *t* statistic; *p* = Probability value; LLCI = Lower limit of the 95% confidence interval; ULCI = Upper limit of the 95% confidence interval; *β* = Standardized coefficient; *R*^2^ = proportion of variance explained. Robust standard errors were used to account for heteroscedasticity.

**Table 7:** Total, direct and indirect effects.

Effect	*B*	*SE*	*t*	*p*	LLCI	ULCI	*β*
Total effect (*c*)	0.09	0.01	7.42	<0.001	0.07	0.11	0.43
Direct effect (*c*’)	0.01	0.02	0.76	0.45	−0.02	0.05	0.06
Indirect total effect	0.08	0.01			0.05	0.10	0.37
Indirect effect via *M*1 (DEP)	0.07	0.01			0.05	0.09	0.34
Indirect effect via *M*2 (ANX)	0.00	0.01			−0.03	0.03	0.00
Indirect effect via *M*3 (STR)	0.00	0.01			−0.02	0.03	0.02

*B* = Unstandardized coefficient; *SE* = Standard error: for direct and total effects, the heteroscedasticity-consistent HC3 correction was used; for indirect effects, *SE* was estimated via bootstrapping; *t* = *t* statistic; *p* = Probability value; LLCI = Lower limit of the 95% confidence interval; ULCI = Upper limit of the 95% confidence interval; *β* = Standardized coefficient. The confidence intervals for the indirect effects were estimated using bootstrapping.

## Data Availability

The datasets generated and/or analyzed during the current study are not publicly available due to ethical and legal restrictions concerning participant confidentiality but are available from the corresponding author on reasonable request and with appropriate institutional approval.

## Abbreviations

The following abbreviations are used in this manuscript:

MBI-GS	Maslach Burnout Inventory—General Survey
DASS-21	Depression Anxiety Stress Scales (21 items)
DASS-42	Depression Anxiety Stress Scales (42 items)
PSS	Paykel Suicide Scale

## References

[B1-ejihpe-16-00049] Arsenault-Lapierre G., Kim C., Turecki G. (2004). Psychiatric diagnoses in 3275 suicides: A meta-analysis. BMC Psychiatry.

[B2-ejihpe-16-00049] Bados A., Solanas A., Andrés R. (2005). Psychometric properties of the Spanish version of Depression, Anxiety and Stress Scales (DASS). Psicothema.

[B3-ejihpe-16-00049] Bartram D. J., Yadegarfar G., Baldwin D. S. (2009a). A cross-sectional study of mental health and well-being and their associations in the UK veterinary profession. Social Psychiatry and Psychiatric Epidemiology.

[B4-ejihpe-16-00049] Bartram D. J., Yadegarfar G., Baldwin D. S. (2009b). Psychosocial working conditions and work-related stressors among UK veterinary surgeons. Occupational Medicine.

[B5-ejihpe-16-00049] Best C. O., Perret J. L., Hewson J., Khosa D. K., Conlon P. D., Jones-Bitton A. (2020). A survey of veterinarian mental health and resilience in Ontario, Canada. The Canadian Veterinary Journal.

[B6-ejihpe-16-00049] Bonde J. P. E. (2008). Psychosocial factors at work and risk of depression: A systematic review of the epidemiological evidence. Occupational and Environmental Medicine.

[B7-ejihpe-16-00049] Bresó E., Salanova M., Schaufeli W. (2006). Síndrome de estar quemado por el trabajo “Burnout” (III): Instrumento de medición *(NTP 732)*.

[B8-ejihpe-16-00049] Cohen J. (1988). Statistical power analysis for the behavioral sciences.

[B9-ejihpe-16-00049] Cordova M. J., Gimmler C., Dibbern A., Duesterdieck-Zellmer K. F. (2025). Career-long skills for personal and professional wellness: A staged developmental model of veterinarian resilience training. Journal of Veterinary Medical Education.

[B10-ejihpe-16-00049] Crane M. F., Phillips J. K., Karin E. (2015). Trait perfectionism strengthens the negative effects of moral stressors occurring in veterinary practice. Australian Veterinary Journal.

[B11-ejihpe-16-00049] Dalum H. S., Tyssen R., Hem E. (2022). Prevalence and individual and work-related factors associated with suicidal thoughts and behaviours among veterinarians in Norway: The NORVET study. BMJ Open.

[B12-ejihpe-16-00049] da Silva C. R., Gomes A. A. D., dos Santos-Doni T. R., Antonelli A. C., da Costa Vieira R. F., da Silva A. R. S. (2023). Suicide in veterinary medicine: A literature review. Veterinary World.

[B13-ejihpe-16-00049] Daza P., Novy D. M., Stanley M. A., Averill P. (2002). The depression anxiety stress scale-21: Spanish translation and validation with a Hispanic sample. Journal of Psychopathology and Behavioral Assessment.

[B14-ejihpe-16-00049] De Witte H. (2005). Job insecurity: Review of the international literature on definitions, prevalence, antecedents and consequences. SA Journal of Industrial Psychology.

[B15-ejihpe-16-00049] Dolan E. D., Mohr D., Lempa M., Joos S., Fihn S. D., Nelson K. M., Helfrich C. D. (2015). Using a single item to measure burnout in primary care staff: A psychometric evaluation. Journal of General Internal Medicine.

[B16-ejihpe-16-00049] Erbe A. M. (2024). Beyond the stethoscope: Exploring mental health issues and suicide risk among veterinarians. Doctoral dissertation.

[B17-ejihpe-16-00049] Felipe A. E. (2025). Salud profesional integral en medicina veterinaria: Problemas psicosociales. Revista de Medicina Veterinaria.

[B18-ejihpe-16-00049] Fonseca-Pedrero E., Pérez de Albéniz A. (2020). Evaluación de la conducta suicida en adolescentes: A propósito de la escala paykel de suicidio. Papeles del Psicólogo.

[B19-ejihpe-16-00049] Fritschi L., Morrison D., Shirangi A., Day L. (2009). Psychological well-being of Australian veterinarians. Australian Veterinary Journal.

[B20-ejihpe-16-00049] Hatch P. H., Winefield H. R., Christie B. A., Lievaart J. J. (2011). Workplace stress, mental health, and burnout of veterinarians in Australia. Australian Veterinary Journal.

[B21-ejihpe-16-00049] Hawton K. (2007). Restricting access to methods of suicide: Rationale and evaluation of this approach to suicide prevention. Crisis.

[B22-ejihpe-16-00049] Hayes A. F. (2022). Introduction to mediation, moderation, and conditional process analysis: A regression-based approach.

[B23-ejihpe-16-00049] Hernández-Esteve I., Zumbado M., Henríquez-Hernández L. A. (2025). Burnout and mental health among veterinarians: The role of self-compassion and associated risk factors. VetRecord.

[B24-ejihpe-16-00049] Instituto Nacional de Estadística (n.d.). Veterinarios colegiados por año y sexo.

[B25-ejihpe-16-00049] Jansen W., Lockett L., Colville T., Uldahl M., De Briyne N. (2024). Veterinarian—Chasing a dream job? A comparative survey on wellbeing and stress levels among european veterinarians between 2018 and 2023. Veterinary Sciences.

[B26-ejihpe-16-00049] Karasek R. A. (1979). Job demands, job decision latitude, and mental strain: Implications for job redesign. Administrative Science Quarterly.

[B27-ejihpe-16-00049] Koutsimani P., Montgomery A., Georganta K. (2019). The relationship between burnout, depression, and anxiety: A systematic review and meta-analysis. Frontiers in Psychology.

[B28-ejihpe-16-00049] Lewis E. G., Cardwell J. M. (2020). The big five personality traits, perfectionism and their association with mental health among UK students on professional degree programmes. BMC Psychology.

[B29-ejihpe-16-00049] Lovibond S. H., Lovibond P. F. (1995). Manual for the depression anxiety & stress scales.

[B30-ejihpe-16-00049] Martínez-Galiano J. M., Martínez-Vázquez S., Peinado-Molina R. A., Hernández-Martínez A. (2024). Validation of the paykel suicide scale and the plutchik suicide risk scale in Spanish women during the perinatal period. Depression and Anxiety.

[B31-ejihpe-16-00049] Maslach C., Jackson S. E., Leiter M. P. (1996). Maslach burnout inventory manual.

[B32-ejihpe-16-00049] Maslach C., Leiter M. P. (2016). Understanding the burnout experience: Recent research and its implications for psychiatry. World Psychiatry.

[B33-ejihpe-16-00049] Maslach C., Schaufeli W. B., Leiter M. P. (2001). Job burnout. Annual Review of Psychology.

[B34-ejihpe-16-00049] Máté M., Várnai C. H., Ózsvári L. (2025). A cross-national study on mental health, psychological distress and suicidal ideation among veterinarians in multiple European countries. Frontiers in Veterinary Science.

[B35-ejihpe-16-00049] McEwen B. S., Tucker P. (2011). Critical biological pathways for chronic psychosocial stress and research opportunities to advance the consideration of stress in chemical risk assessment. American Journal of Public Health.

[B36-ejihpe-16-00049] Menon N. K., Shanafelt T. D., Sinsky C. A., Linzer M., Carlasare L. J., Brady K. J., Stillman J. M., Trockel M. T. (2020). Association of physician burnout with suicidal ideation and medical errors. JAMA Network Open.

[B37-ejihpe-16-00049] Milner A., Witt K., LaMontagne A. D., Niedhammer I. (2018). Psychosocial jobstressors and suicidality: A meta-analysis and systematic review. Occupational and Environmental Medicine.

[B38-ejihpe-16-00049] Moses L., Malowney M. J., Boyd J. W. (2018). Ethical conflict and moral distress in veterinary practice: A survey of North American veterinarians. Journal of Veterinary Internal Medicine.

[B39-ejihpe-16-00049] Nett R. J., Witte T. K., Holzbauer S. M., Elchos B. L., Campagnolo E. R., Musgrave K. J., Carter K. K., Kurkjian K. M., Vanicek C. F., O’Leary D. R., Pride K. R., Funk R. H. (2015). Risk factors for suicide, attitudes toward mental illness, and practice-related stressors among US veterinarians. Journal of the American Veterinary Medical Association.

[B40-ejihpe-16-00049] Netterstrøm B., Conrad N., Bech P., Fink P., Olsen O., Rugulies R., Stansfeld S. (2008). The relation between work-related psychosocial factors and the development of depression. Epidemiologic Reviews.

[B41-ejihpe-16-00049] Neubauer V., Dale R., Probst T., Pieh C., Janowitz K., Brühl D., Humer E. (2024). Prevalence of mental health symptoms in Austrian veterinarians and examination of influencing factors. Scientific Reports.

[B42-ejihpe-16-00049] Nunnally J. C. (1978). Psychometric theory.

[B43-ejihpe-16-00049] Osca A., Barrado J., Millán L. (2024). Validation of the burnout assessment tool-core symptoms in Spanish veterinarians, sex invariance, and cutoff points. Frontiers in Veterinary Science.

[B44-ejihpe-16-00049] Paykel E. S., Myers J. K., Lindenthal J. J., Tanner J. (1974). Suicidal feelings in the general population: A prevalence study. The British Journal of Psychiatry.

[B45-ejihpe-16-00049] Peixoto M. M. (2025). Suicide risk in veterinary professionals in Portugal: Prevalence of psychological symptoms, burnout, and compassion fatigue. Archives of Suicide Research.

[B46-ejihpe-16-00049] Perret J. L., Best C. O., Coe J. B., Greer A. L., Khosa D. K., Jones-Bitton A. (2020). Prevalence of mental health outcomes among Canadian veterinarians. Journal of the American Veterinary Medical Association.

[B47-ejihpe-16-00049] Peterson C., Stone D. M., Marsh S. M., Schumacher P. K., Tiesman H. M., McIntosh W. L., Lokey C. N., Trudeau A.-R. T., Bartholow B., Luo F. (2018). Suicide rates by major occupational group—17 states, 2012 and 2015. MMWR Morbidity and Mortality Weekly Report.

[B48-ejihpe-16-00049] Pinazo-Clapés C., Redondo R., Checa I., Pinazo-Hernandis S., Sales A., Pons J. (2025). Screening of suicidal ideation in nursing homes: Validation of the paykel scale in older adults. Aging & Mental Health.

[B49-ejihpe-16-00049] Podpecan O., Hlebec V., Kuhar M., Kubale V., Jakovac-Strajn B. (2025). Predictors of burnout and well-being among veterinarians in Slovenia. Veterinary Sciences.

[B50-ejihpe-16-00049] Pohl R., Botscharow J., Böckelmann I., Thielmann B. (2022). Stress and strain among veterinarians: A scoping review. Irish Veterinary Journal.

[B51-ejihpe-16-00049] Ruiz F. J., Martín M. B., Falcón J. C., Odriozola-González P. (2017). The hierarchical factor structure of the Spanish version of depression anxiety and stress scale-21. International Journal of Psychology and Psychological Therapy.

[B52-ejihpe-16-00049] Salanova M., Schaufeli W. B., Llorens S., Peiró J. M., Grau R. (2000). Desde el “burnout” al “engagement”: ¿una nueva perspectiva?. Revista de Psicología del Trabajo y las Organizaciones.

[B53-ejihpe-16-00049] San Martín A., San Martín P., Míguez-Santiyán M. P., Soler F., Pérez-López M. (2023). Prevalence of burnout syndrome among veterinarians in Spain. Journal of the American Veterinary Medical Association.

[B54-ejihpe-16-00049] Schaufeli W. B., Leiter M. P., Maslach C., Jackson S. E., Maslach C., Jackson S. E., Leiter M. P. (1996). Maslach burnout inventory—General survey. The maslach burnout inventory—Test manual.

[B55-ejihpe-16-00049] Schunter N., Bahramsoltani M., Böhler L., Glaesmer H. (2025). Study-related predictors for depression, suicidal ideation and suicide risk in German veterinary medical students. Healthcare.

[B56-ejihpe-16-00049] Schwerdtfeger K. A., Glaesmer H., Bahramsoltani M. (2024). High overcommitment and low reward as potential predictors for increased depressive symptoms, suicidal ideation, and suicide risk in German veterinarians. PLoS ONE.

[B57-ejihpe-16-00049] Seidler A., Thinschmidt M., Deckert S., Then F., Hegewald J., Nieuwenhuijsen K., Riedel-Heller S. G. (2014). The role of psychosocial working conditions on burnout and its core component emotional exhaustion: A systematic review. Journal of Occupational Medicine and Toxicology.

[B58-ejihpe-16-00049] Siegrist J. (2002). Effort-reward imbalance at work and health. Historical and current perspectives on stress and health.

[B59-ejihpe-16-00049] Smith J., Hawgood J. (2025). Suicide prevention for the veterinary profession—A preliminary investigation to explore veterinarians’ perceptions of ASIST training for their profession. Frontiers in Veterinary Science.

[B60-ejihpe-16-00049] Tomasi S. E., Fechter-Leggett E. D., Edwards N. T., Reddish A. D., Crosby A. E., Nett R. J. (2019). Suicide among veterinarians in the United States from 1979 through 2015. Journal of the American Veterinary Medical Association.

[B61-ejihpe-16-00049] West C. P., Dyrbye L. N., Satele D. V., Sloan J. A., Shanafelt T. D. (2012). Concurrent validity of single-item measures of emotional exhaustion and depersonalization in burnout assessment. Journal of General Internal Medicine.

[B62-ejihpe-16-00049] Witte T. K., Spitzer E. G., Edwards N., Fowler K. A., Nett R. J. (2019). Suicides and deaths of undetermined intent among veterinary professionals from 2003 through 2014. Journal of the American Veterinary Medical Association.

[B63-ejihpe-16-00049] Yip P. S. F., Caine E., Yousuf S., Chang S. S., Wu K. C. C., Chen Y. Y. (2012). Means restriction for suicide prevention. The Lancet.

